# ThreatSim: A novel stimuli database of threatening and nonthreatening image pairs rated for similarity

**DOI:** 10.3758/s13428-025-02906-w

**Published:** 2025-12-10

**Authors:** Andras N. Zsido, Michael C. Hout, Eben W. Daggett, Julia Basler, Otilia Csonka, Bahtiyar Yıldız, Marko Hernandez, Bryan White, Botond Laszlo Kiss

**Affiliations:** 1 Institute of Psychology, University of Pécs, 6. Ifjusag Street, Pécs, Baranya H 7624 Hungary; 2 Contemporary Challenges Research Centre, University of Pécs, Pécs, Hungary; 3 Psychology Department, New Mexico State University, Las Cruces, NM USA; 4 Kinesiology Department, New Mexico State University, Las Cruces, NM USA; 5 Szentágothai Research Centre, University of Pécs, Pécs, Hungary

**Keywords:** Image database, Visual search, Threat, Animal, Object, Visual similarity

## Abstract

Researchers often require validated and well-rounded sets of image stimuli. For those interested in understanding the various visual attentional biases toward threatening stimuli, a dataset containing a variety of such objects is urgently needed. Here, our goal was to create an image database of animate and inanimate objects, including those that people find threatening and those that are visually similar to them but are not considered threatening. To do this, we recruited participants (*N* = 77) for an online survey in which they were asked to name threatening objects and try to come up with a visually similar counterpart. We then used the survey results to create a list of 32 objects, including eight from each crossing of threatening versus nonthreatening and animate versus inanimate. We obtained 20 exemplar images from each category (640 unique images in total, all copyright-free and openly shared). An independent sample of participants (*N* = 191) judged the similarity of these images using the spatial arrangement method. Data were then modeled using multidimensional scaling. Our results present modeling outcomes using a “map” of animate and inanimate objects (separately) that spatially conveys the perceived similarity relationships between them. We expect that this image set will be widely used in future visual attention studies and more.

## Introduction

**Fig. 1:**
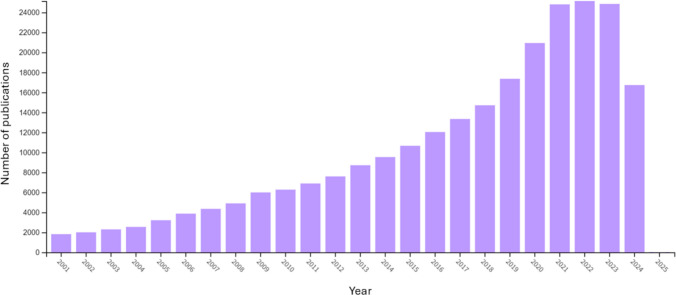
The number of papers published in the field of threat research in visual perception and attention according to our Web of Science search (using the search terms “*Threat, Visual Perception, Attention [All Fields] and Threat [Should - Search within topic] and Threat Perception [Should - Search within topic] and Threat Detection [Should - Search within topic] and Attentional Bias [Should - Search within topic]*”) in the past 25 years

Attention to (and perception of) threats has long been a prominent area of visual cognition research, with the first experiments conducted nearly four decades ago. The field is still thriving today, with the number of papers increasing dramatically in recent years. We conducted a Web of Science search (only a crude type, not a systematic review) with the intention of giving a sense of the overall upward trend of publishing in this area. We found 255 papers in 1983, 777 in 1993, 2,299 in 2003, 8,718 in 2013, and 24,853 in 2023 (see Fig. 1 for the past 25 years). This steep rise is at least partly due to a methodological paradigm shift that has taken place in recent years, initiated by a review (McNally, 2018), and since followed on by a large number of other projects (e.g., Bonin et al., 2014; Hedger et al., 2019; Lazarević et al., 2020; Loucks et al., 2023; Quinlan, 2013; Song & Wolfe, 2024; Zsido et al., 2024). This collective body of work demonstrates that there are a number of common shortcomings in previous research that call into question issues of stimulus control and the reliability and replicability of prior results, preventing us from drawing appropriate conclusions. Some authors have argued (e.g., Quinlan et al., 2017; Zsido et al., 2019, 2024) that the stimuli used in prior studies are problematic (for a variety of reasons discussed below). Although there are a number of image databases available today, these only partially address the needs of researchers in the field, and their limitations often restrict the research paradigms in which they are used.

### Current picture databases

Early databases, such as the International Affective Picture System (IAPS; Lang et al., 1997), are of limited use because the images have a low resolution by modern standards, and/or the images included are often copyrighted, making it difficult for researchers to find and use them (Deák et al., 2010; Grimaldos et al., 2021) and preventing researchers from being able to display veridical representations of experimental procedures in their publications. This not only is contrary to modern open science norms, but also discourages replication of past research, as obtaining the same images used in previous research can be a tedious task. Furthermore, such images are not versatile enough for threat research because they include a very narrow range of different categories of threats (Dan-Glauser & Scherer, 2011; Goodman et al., 2016), such as snakes or guns.

This limitation leads us to another dilemma: Using the images provided by the dataset limits the scope of the research to a handful of categories, ultimately preventing us from generalizing the results to other threats (Pakai-Stecina et al., 2024; Zsido et al., 2019). Obtaining new images from the Internet to supplement those provided by the stimulus dataset is problematic because Internet-obtained images may not properly match the original ones on key confounding variables such as visual characteristics, complexity, and valence ratings (Bolfing et al., 2008; Donderi & McFadden, 2005; Niu et al., 2012; Onie & Most, 2021). Because each researcher tends to add their own images to an existing database (which are seldom shared thereafter), such cobbled-together image sets are unlikely to be compatible with each other, and thus it will be unclear whether the obtained results are specific to the image set or are truly generalizable (Anderson et al., 2015; Coelho et al., 2010; Humphrey et al., 2012). Mixing existing databases leads to similar problems, as they are rarely compatible with each other in terms of picture characteristics (e.g., size, quality, complexity, background), content (e.g., number, size, and visibility of objects, layout, arrangement), and rating dimensions (e.g., pictures rated as good or bad, positive or negative, threatening or nonthreatening).

Although novel databases have been created to be mindful of these concerns, they are usually designed to meet the needs of a particular research project or laboratory. As a result, they often solve only the problems of that research group (or project), and address only a subset of the shortcomings of existing databases. For example, while existing databases often do provide high-quality images controlled for low-level visual features, they typically still retain the background surrounding the threat item (Kurdi et al., 2017; March et al., 2017). Pictures without background, such as those in the Massive Memory Database (Brady et al., 2008; Hout et al., 2014), are ideal for studying (for instance) how visual attention can be directed toward certain categories of items (uncontaminated by background content), and a comparable set is crucial for researchers to be able to study visual attention to threats in complex visual scenes (Rádlová et al., 2019; Zsido et al., 2019, 2024). Further, not only are the numbers of available categories in extant databases still limited (Dan-Glauser & Scherer, 2011; Grimaldos et al., 2021; Marchewka et al., 2014; Polák et al., 2020), but they rarely ensure that both animate and inanimate items are available in at least approximately equal numbers. This is the case despite the fact that studies on the effects of threatening stimuli using only animal stimuli have assumed that they had an evolutionary advantage in processing over modern objects (LoBue & Matthews, 2014; Öhman et al., 2012)—a claim that has been challenged by more recent studies (Blanchette, 2006; Fox et al., 2007; Subra et al., 2017; Zsido et al., 2018).

Most importantly, new databases may fail to introduce major paradigm-shifting innovations, because they have built upon and patched the shortcomings of previous databases. An example is the introduction of mid-level visual features—the conjunction of elementary features, i.e., shapes and partial geometries of objects (Peirce, 2015)—the role of which has been widely reported in threat research (Coelho et al., 2023; Kennett & Wallis, 2019; Larson et al., 2007, 2009; Pakai-Stecina et al., 2024; Van Strien et al., 2016; Zsido et al., 2022). To our knowledge, no existing database has controlled for such features. This can be done simply by obtaining neutral images that are nonthreatening but visually similar to threats, thus creating paired categories, for example, snakes (threatening) and caterpillars (nonthreatening but visually similar to snakes).

Another problematic common practice is that researchers typically first decide which categories to include in these databases and then ask participants to rate the collected images (Dan-Glauser & Scherer, 2011; Grimaldos et al., 2021; Kurdi et al., 2017; Lang et al., 1997). This could be problematic in that researchers may only feel comfortable relying on categories that have been used in past work, or may exhibit other biases in stimulus selection. A solution to this is crowdsourcing, that is, involving lay people in stimulus selection by asking them what they think is threatening (and what they think is visually similar but nonthreatening). Inclusion of wider viewpoints could improve the validity of these databases and thus the reliability of the results of research conducted using them.

With all of these concerns in mind, our goal in the present study was to create a novel stimulus database for visual threat research that

1. Includes both animate and inanimate stimuli
2. Whose categories are derived by layperson input and
3. Which have been rated for visual similarity.

### The importance of similarity

The visual similarity of the stimuli used in experiments studying attention is crucial, as it can affect the ease and probability of success, particularly during tasks like visual search (Duncan & Humphreys, 1989). In the context of this study, “visual similarity” refers to the perceived similarity in appearance, as judged by naïve observers. In other words, two objects are considered visually similar if participants judge them to be similar in terms of shape, form, or other visible features. Similarity modeling provides a reliable means of understanding and quantifying how people perceive relationships between objects (Hout et al., 2016). Unlike complex computational methods, human ratings are straightforward, and have been fundamental to the development of models of similarity perception (Nosofsky, 1986; Shepard, 2004). Indeed, human judgments can integrate both physical and semantic features of objects, providing a comprehensive perspective that computational methods may miss (Hout et al., 2016). Multidimensional scaling (MDS), a statistical technique that maps perceived similarities onto a spatial representation, can be used to explore and represent these relationships between objects according to similarity ratings obtained from human participants (Hout, Papesh et al., 2013b). By representing items in a spatial configuration, MDS aims to reflect the perceived relational “distances” between items as accurately as possible using group-level aggregated dissimilarity matrices. This method can also help uncover the underlying dimensions that were appreciated when raters provided their data, making it a valuable tool in understanding perceptual or conceptual spaces.

One particularly promising method for obtaining similarity data is the spatial arrangement method (SpAM), in which participants arrange items on a screen according to how similar they are. Specifically, they are instructed to arrange the items in space such that the distance between any two items reflects the participant’s perceived similarity of the pair, with closer spacing denoting pairs that are perceived as similar, and proportionately farther placement between items indicating higher perceived dissimilarity. SpAM is an efficient and effective approach to eliciting similarity ratings (Hout et al., 2014; Hout, Goldinger, et al., 2013a; Hout & Goldinger, 2016). Unlike traditional methods that require participants to compare pairs of objects (Daggett & Hout, 2025), SpAM allows participants to arrange multiple object sets simultaneously based on perceived similarities between a group of items. This method is especially effective for large datasets (see Hout et al., 2018), reducing the time required for data collection while maintaining high-quality results. By providing a visual representation of how items are related, SpAM facilitates the creation of a proximity matrix that can be used for further analysis. A number of prior studies used these methods to assess similarities between objects. For instance, SpAM was used to examine the similarity structure of real-world scenes (Berman et al., 2014; Coburn et al., 2019; Karadan et al., 2015), while another study (Horst & Hout, 2016) used it to collect similarity ratings for large sets of novel objects across different categories, demonstrating the efficiency and effectiveness of the method in large-scale research.

### The current study

In the present study, we attempted to create a novel image database that addresses the limitations of previous ones and thus can be used in a variety of threat research paradigms. We sought to obtain image categories based on the input of naïve participants who were blind to the purpose of the database. On the basis of their responses and our discussion, we conducted an online search and compiled a final list of eight categories, according to the four classes (“threatening” animals and objects, and “nonthreatening” but visually similar animals and objects), with 20 exemplars in each category. This resulted in a total of 640 images (4 × 8 × 20). In addition, our goal was to create a database containing a large number of categories of threatening and nonthreatening objects, to include animate and inanimate items, to have pairs matched for appearance, and to have categories represented by multiple exemplars (to allow for natural variance within the category). We then sought to include measures of item similarity for multiple exemplars across the categories. To maximize the potential of the database, we sought to include only images without backgrounds and, importantly, images that are freely available and can be easily utilized by researchers.

Crucially, our goal was not to define or model the specific features contributing to judgments of visual similarity, but rather to provide a tool that quantifies perceived similarity relationships across a wide range of threat-relevant images. This will allow researchers to select stimuli that are matched in terms of perceived similarity, which is a key factor in controlling for visual confounds in threat-related experiments. Although this study does not examine differences between animate and inanimate threats, we aimed to ensure that both categories were adequately represented in the database. This design choice reflects the importance of animacy as a theoretically relevant dimension in threat perception research. By balancing the number of animate and inanimate categories, we have made it easier for future studies to explore potential processing differences between these groups under controlled conditions.

## Methods

### Participants

**Fig. 2:**
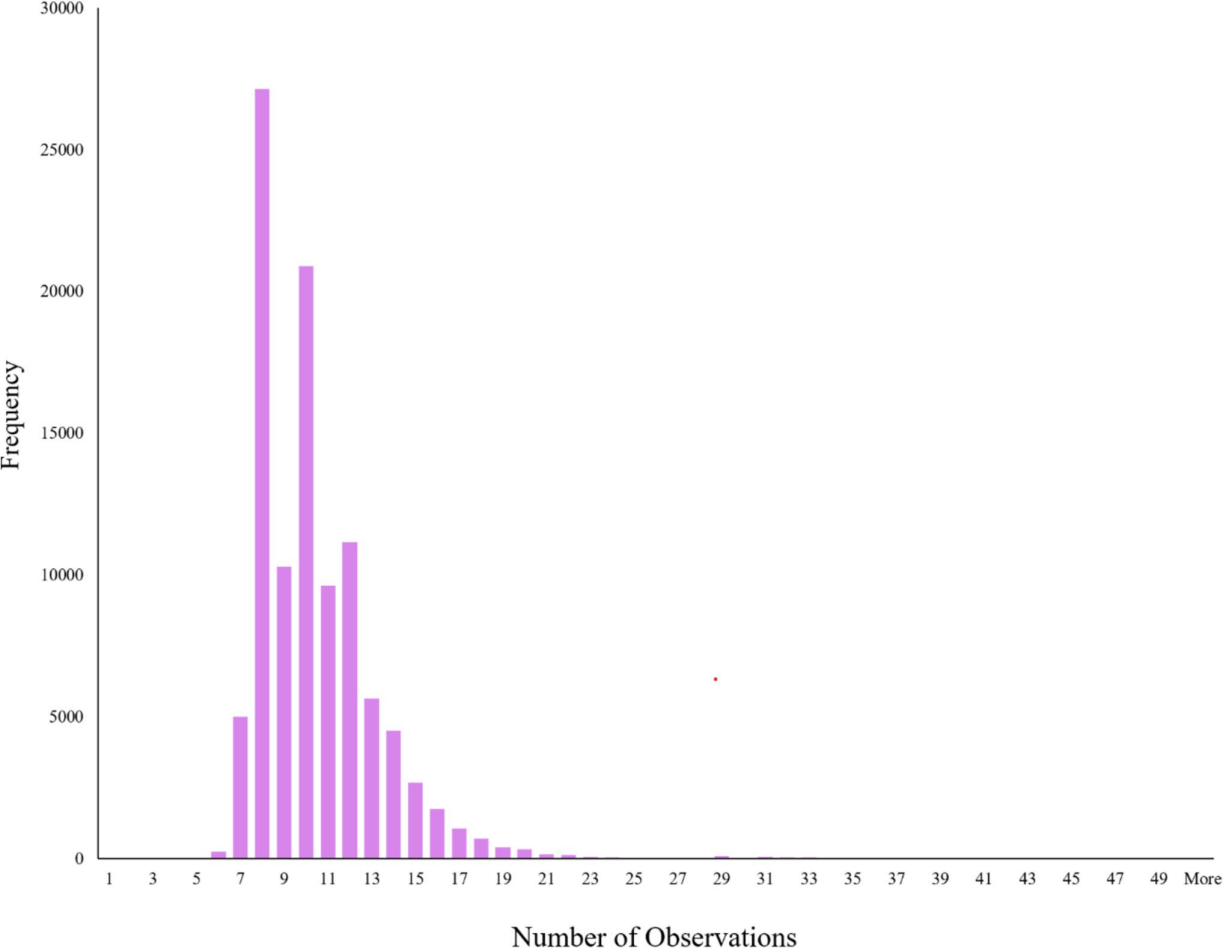
The number of observations per pair of stimuli in the dataset we collected

A priori procedures for determining sample size in MDS do not exist, given the nature of the approach. Therefore, to ensure that we collected an adequate dataset, instead of aiming for a predetermined number of participants, we aimed to acquire multiple comparisons for each possible pair of the 640 images. Presenting all the stimuli on the screen at one time is clearly not possible, so instead we utilized a multi-trial version of SpAM that presents subsets of stimuli on each trial. Our goal was to provide enough quasi-random sets of stimuli that each pair of items were presented together a minimum of three times (per site). To do this, we used an algorithm (MacDonald et al., 2019) to generate the quasi-random selection of items and to predetermine all trial sequences that were necessary. This resulted in 950 trials for inanimate and 950 for animate stimuli (per site). Figure 2 shows the distribution of the number of pairwise comparisons in our data. Nearly all pairs of stimuli received at least three comparisons, and the number of comparisons approximates a normal distribution with a positive skew. Additionally, it should be noted that MDS spaces comprising larger sets of items tend to result in a better-quality MDS space relative to those with fewer items (Hout et al., 2018). This is because the placement of each item in MDS space is determined by the reported similarities between it and every other item in the space. Each point in MDS space therefore has more data “pulling it into place” as the stimulus set grows. Given the very large number of stimuli in our set, even a single similarity score based on a small number of observations is to some degree protected by the many other similarity scores that ultimately determine its location in MDS space.

A total of 191 students participated in this study, 110 from the University of Pécs and 81 from the New Mexico State University. All participants reported normal or corrected-to-normal vision, and understood the study instructions. Our research was approved by the Hungarian United Ethical Review Committee for Research in Psychology (approval number 2023-107) and the Institutional Review Board of New Mexico State University (Protocol number 2308075078) and was carried out in accordance with the Code of Ethics of the World Medical Association Declaration of Helsinki. All participants provided written informed consent. Participants at the University of Pécs were compensated for their efforts with payment of 5,000 HUF (approximately 15 USD). Participants from New Mexico State University were volunteers from classes who received partial course credit for participating. The study was preregistered at OSF: https://osf.io/8m6cn.

### Stimuli set

First, we created an online survey using Google Forms and asked respondents (*N* = 77) to list the names of categories (of objects and animals) that they and others would find threatening, and also to provide visually similar nonthreatening pairs. The sample size for the initial survey was not determined via a formal power analysis, as the purpose of this phase was exploratory and qualitative—aimed at generating a wide range of potential categories rather than testing a hypothesis. We continued recruitment until the responses yielded a sufficiently broad and diverse set of candidate threat categories, and we observed saturation in the types of threats named by participants. Specifically, the instructions were to:

...*write inanimate objects* [or animals] *which can be perceived as threatening according to you or others, as well as objects which look similar to them due to some physical attributes. For example, a banjo would have a similar shape to a frying pan. There are no right or wrong answers, please write your thoughts without extensive thinking, following your instincts. The* *number* *of answers you can provide is not limited; please write as many objects that come to your mind and fit the given question*.

We sought to populate four overall classes of items, crossing animate versus inanimate and threatening versus nonthreatening (but visually similar), with a goal of generating a large number of visually distinct categories. We then identified the threatening objects that were most frequently named by respondents and paired them with visually similar categories. This was followed by a discussion among the authors of this manuscript to curate the final list of objects. This was necessary because some threatening objects named by the respondents were semantically and visually very similar to each other (e.g., tiger and lion), so using them as separate categories would have reduced the diversity of categories (or the heterogeneity of visual features) included overall. In some such cases, we decided to merge the two categories and split the number of exemplars of each within the category. Furthermore, the visually similar nonthreatening pairs of objects varied considerably across respondents. We therefore decided again to use multiple subcategories and discussed among ourselves which ones to include (e.g., we matched grenades with Christmas tree ornaments, lime juice containers, and perfume bottles). We chose to include eight categories to balance the desire for variety with the need for a manageable number of images in the subsequent MDS phase. Table 1 presents a list of objects we selected, broken down by image category.

**Table 1:** Object categories selected in our study, broken down by class

Threatening	Nonthreatening
**Animate**	
Snake	Caterpillar, worm
Wolf, hyena	Dog
Tiger, lion	Cat
Scorpion	Stink bug, beetle
Spider	Ant
Wasp, hornet	Bee, fly
Shark	Whale, dolphin
Alligator, crocodile	Lizard, turtle
**Inanimate**	
Pistol	Hairdryer, toy gun
Knife, cleaver	Kitchen utensils (butter knife, pie spoon)
Long gun	Paintball gun, water gun
Grenade	Christmas tree ornaments, lime juice container, perfume bottles
Syringe	Knitting/sewing needle
Taser	Electric razor, mobile recorder
Baseball bat, baton	Rolling pin, bowling pin, umbrella
Landmine	Weights

After we obtained our list of categories, we sourced color images from the Internet that were free to use and could be shared for noncommercial purposes. The search criteria were to find examples

1. That were shown on white backgrounds (or with backgrounds that could be cleanly removed),
2. That were photographed preferably from a side view, and
3. Where only the object was visible in the image (before or after background removal).

We continued the search until we obtained eight categories per class (again, threatening animals and objects, nonthreatening but visually similar animals and objects) and 20 exemplars per category. This resulted in a total of 640 images (4 × 8 × 20). The images were then rescaled and cropped to a uniform size and placed inside a 200-pixel-diameter circle (therefore, in this database, real size is not a factor). All pictures are available from the OSF site of the project: https://osf.io/cmtdw/.

### Procedure

Participants completed two blocks of similarity rating trials using SpAM: one set involving animate stimuli only and another involving inanimate stimuli only (presented in counterbalanced order). This was necessary to reduce the likelihood of coarse, category-driven clustering (e.g., animals versus tools); animate and inanimate objects were rated separately. Different trial sequences were generated for each stimulus set. The task structure, instructions, and method of analysis were identical for both blocks. Each block was analyzed independently using MDS to map the perceived similarity structure within each stimulus class separately.

**Fig. 3:**
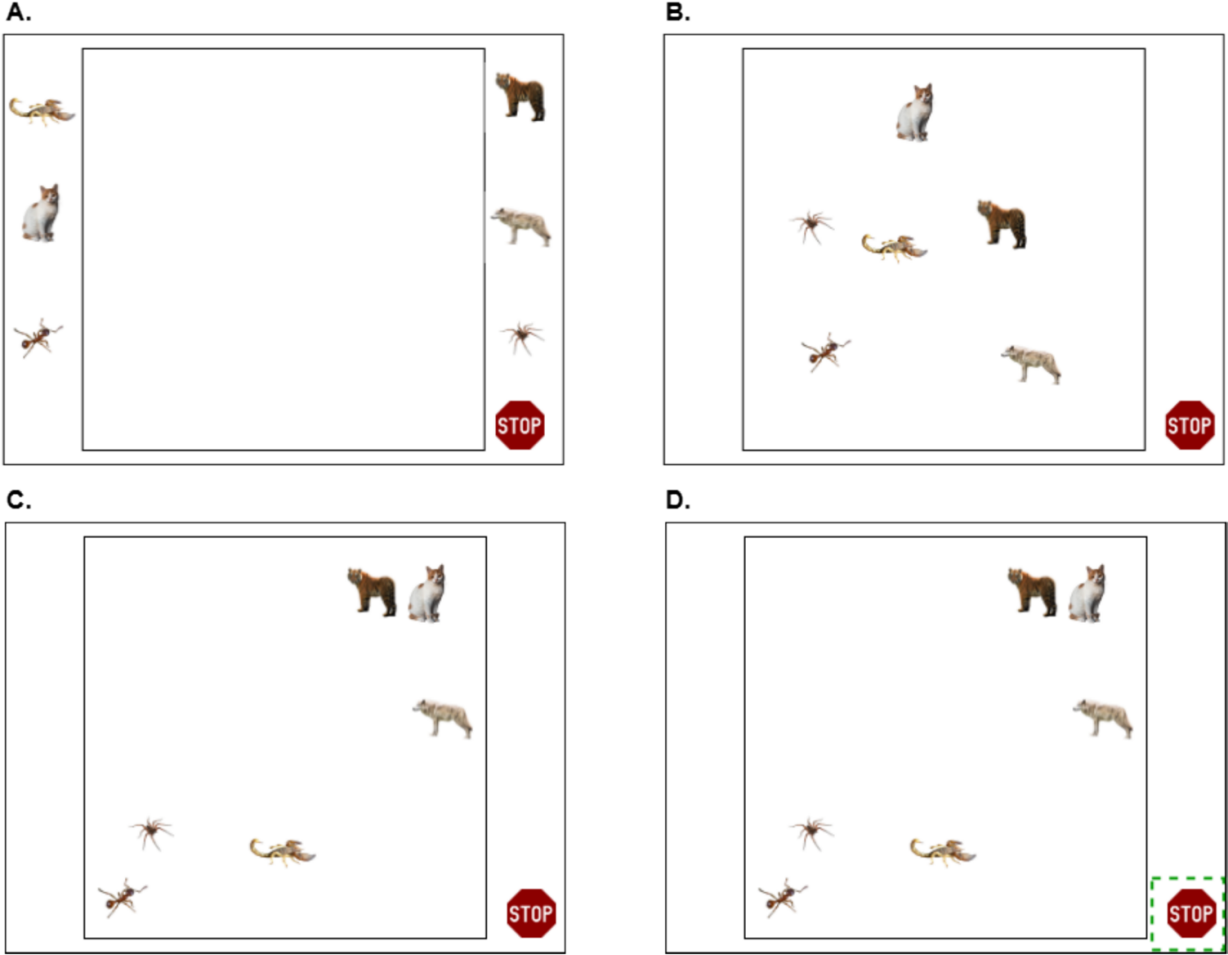
A simplified time-lapse depiction of a typical SpAM trial in this study. At the onset of a trial (**A**), objects are arranged on either side of the “arena.” Participants then drag and drop all objects into the arena with rough similarity relationships (**B**). Participants spend some amount of time “tuning” the arrangement of objects within the space until they achieve an arrangement that satisfies the task (**C**). To end a trial, participants click on a stop sign icon in the bottom right of the screen (**D**)

We collected similarity ratings among the category exemplars—later scaled using multidimensional scaling—in order to provide a similarity model of the exemplars that could be used by researchers for stimulus selection. Participants rated the images using the SpAM (Fig. 3), which was programmed in E-Prime, and we used 32-inch 4K LG monitors (resolution 3,840 × 2,160) for data collection. We used a greedy algorithm to minimize the number of trials required, to ensure that every item would be compared to every other item several times (MacDonald et al., 2019); our SpAM trials were thus pre-populated with a quasi-random selection of items. Each participant conducted as many SpAM trials as they could in the allotted time period, and the next participant merely picked up where the previous one left off. This was done to accommodate the variability in SpAM organization time between participants, with some taking longer to rate the stimuli than others.

The algorithm parameters were set such that we would obtain a minimum of three comparisons between each pair of exemplars. The algorithm’s output indicated that we needed 950 trials each for inanimate and animate stimuli. Participants rated pictures of animals and inanimate objects separately, in order to place more emphasis on smaller individualizing features and to prevent the categories “animate” and “inanimate” from overshadowing the judgments (i.e., leading to uninformative “clumping” whereby all the animate items were on one side of the MDS space and the inanimate ones were on the other).

Upon arrival at the lab, each participant was shown a short tutorial video explaining the trial and task procedure. (The video can be found on the OSF site of the project: https://osf.io/cmtdw/.) They were then free to ask questions or watch the video again if they felt the need. On each trial, 24 images appeared on the screen, initially positioned outside a central square area known as the “arena.” The dimensions of the arena were 2,160 × 2,160 pixels, and it was bounded by a bold black border. Participants were instructed to drag and drop all of the images into the arena, arranging them so that those they perceived as visually similar were placed close together and those they perceived as dissimilar were placed farther apart. There was no time limit for completing a trial. Participants could indicate that they had finished arranging the objects in the arena by clicking a “stop” button in the lower right corner of the screen.

The trial could only be completed when all objects had been moved into the arena. Once all objects had been moved and the participants clicked on the stop sign, a text was presented asking whether they had finished arranging the arena, needed more time, or wanted to start over. If participants indicated that they were done, the data (i.e., the Euclidean distances [measured in pixels] between each pair of images on the screen) were recorded, and participants moved on to the next trial. If they indicated that they needed more time, they were directed back to their SpAM arrangement. If they indicated that they wanted to start over, all items were moved back to their original positions outside of the arena, and the trial began anew. Each participant rated both inanimate and animate objects for 20 min each, in counterbalanced order. Thus, the experiment lasted approximately 1 h (accounting for breaks, time for informed consent, and so forth).

### Data analyses

As we were interested in the internal similarity structures of animate and inanimate objects rather than direct comparison between them, and as they were rated in separate blocks, we conducted two independent MDS analyses—one for animate categories and one for inanimate categories.

The data recorded from each SpAM trial were the Euclidean distances (measured in pixels) between each pair of images on the screen. This procedure is standard, and is well documented in papers such as Hout, Goldinger et al. (2012a), and Goldstone (1994). For each stimulus set (animate and inanimate), the raw dissimilarity matrices obtained from the SpAM were first normalized and then averaged across participants to produce a group-level dissimilarity matrix. This aggregation process controls for differences in scaling between participants (e.g., some participants using generally larger or smaller distances) and produces a consensus representation of perceived similarity. MDS was then applied to these group-level matrices to produce the configurations shown in the results section. The coordinates are relative; absolute distances cannot be interpreted directly across configurations. However, within each MDS space, smaller distances indicate greater perceived similarity between items.

R scripts used to conduct the analysis, the raw dissimilarity matrixes (tables that quantify how different each pair of images was perceived by participants), MDS coordinates for each of the 10 dimensional solutions (for each image category, we created multiple MDS solutions with increasing dimensionality from 1 to 10), MDS plots, scree plots of dimensionality versus stress (these values indicate the extent to which the spatial model accurately represents the original similarity ratings, with lower stress indicating better fit), spreadsheets of pairwise Euclidean distance, and spreadsheets detailing the distance of each image from the centroid (the centroid is the “center of mass” of the space, emphasizing the density of objects in each area of the space rather than using a simple geometric calculation of center) of the MDS space can be found on the OSF page of the project: https://osf.io/cmtdw/

## Results

### Open access materials

All data discussed in the analysis section can be found in the Open Science Framework page of the study (https://osf.io/cmtdw/). The page houses a downloadable zip file with the following key content:

1. A spreadsheet containing the database of threat stimuli similarity data,
2. The R script utilized to conduct the analysis,
3. A folder containing all raw input data for the R script in .csv format, and
4. A folder containing all outputs from the R script in. csv format that were used to construct the database.

### MDS algorithm

For each of the 32 threat image categories (16 inanimate and 16 animate images), we used Kruskal’s nonmetric multidimensional scaling (MDS) (Kruskal, 1964) to derive a series of MDS coordinate solutions with successively increasing dimensionality (up to a dimensionality of 10). Kruskal’s stress formula was used to calculate a stress metric for each of the 10 dimensional solutions within each of the categories. The MDS analysis was conducted in R, utilizing a script that takes the aggregated similarity data collected via SpAM as input and iteratively runs the data through the MDS program at dimensionality of 1 through *n*, with the experimenter choosing the value of *n* as the maximum dimensionality to be utilized. With each successive iteration of the process, the script produces a coordinate set, a measure of stress, measures of centrality, and a metric called “uniqueness” (detailed below) for each item in the set. Centrality is a measure of the distance of an object from the centroid relative to the other objects in the MDS space. This can be interpreted as a type of “prototypicality,” where objects closer to the centroid are likely more representative of the other objects in the space. Uniqueness is a measure of how visually distinct an item is from others in the same group, where objects that have large average pairwise distances to other objects in the space can be said to be more unique.

### Dimensionality of the MDS space

A traditional methodology for determining the proper dimensionality for the MDS solution is to analyze the reduction in stress exhibited as the dimensionality of the solution is increased. Such data can be visualized in a scree plot. We created a scree plot for each stimulus class, as well as one for the aggregate analysis of animate and inanimate objects. These can be found on the study’s OSF site, with 34 plots in total. A typical heuristic approach for analyzing the scree plot involves the subjective identification of an “elbow” in the often monotonically decreasing stress value as a function of dimensionality. This elbow represents a point at which an increase in dimensionality represents a diminishing return in the reduction of stress (Kruskal & Wish, 1978). Other approaches for determining dimensionality include the use of Bayesian techniques (Lee, 2001) to more objectively determine an optimal trade-off between lower, more interpretable dimensionality and stress.

We present the key findings in figures. To interpret these figures, some guidelines should be mentioned. Each axis represents a dimension of perceived visual similarity from the resulting MDS space. Rather than being labeled with fixed features, these dimensions reflect emerging properties based on how people judged similarity during the SpAM task. The coordinate values on each axis are arbitrary in terms of scale and direction. Having a negative or positive value simply indicates a position relative to the geometric center of the MDS space, and does not imply any inherent quality. Objects that are close to one another in MDS space were judged by participants to be visually similar, while those farther apart were perceived as more visually dissimilar.

### Classification of item prototypicality (centrality)

**Fig. 4:**
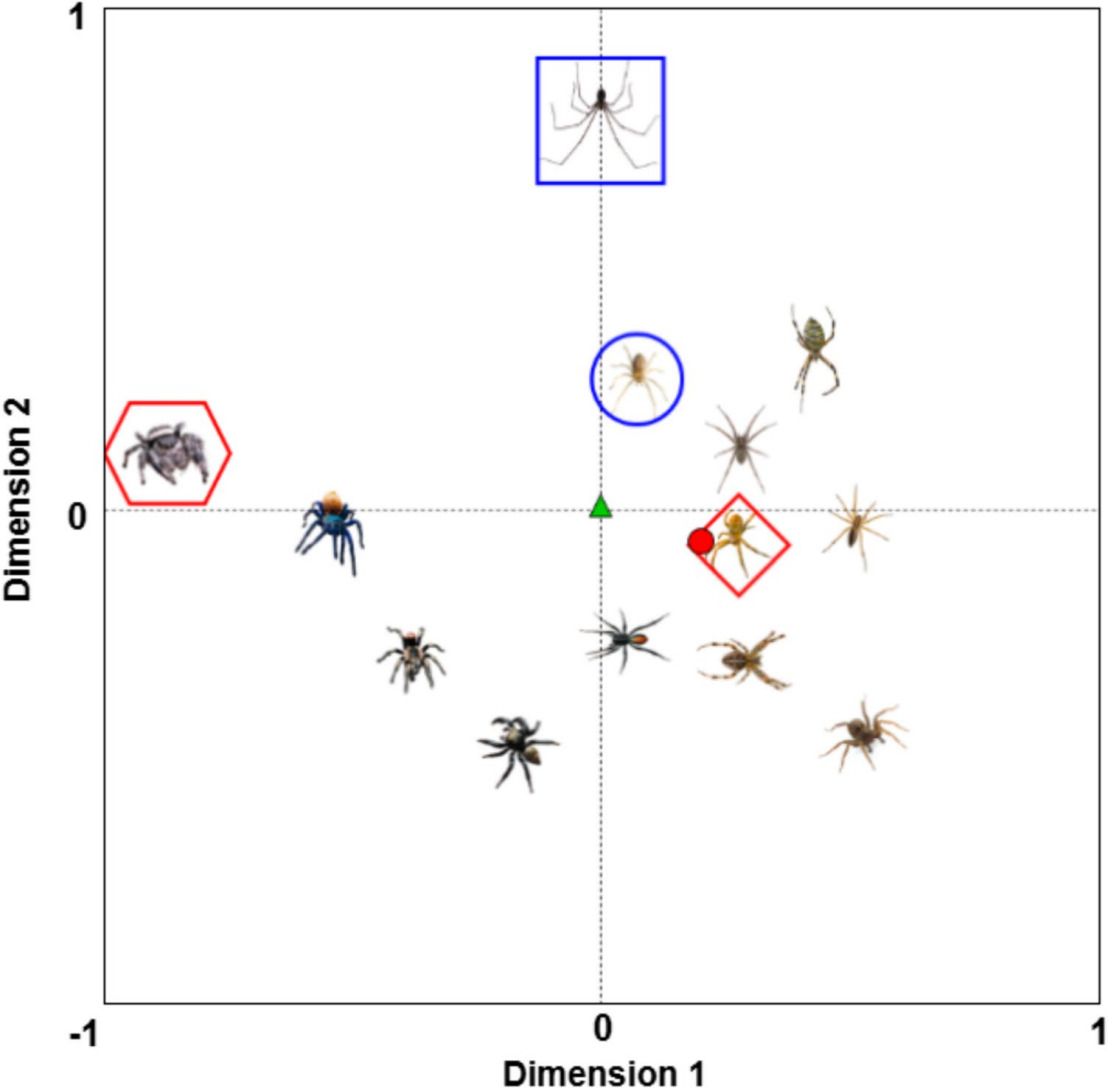
The first two dimensions of an example MDS space of spider images. Both the geometric center (green triangle) and the centroid (red circle) are depicted for visualization purposes. From the visual, it is possible to see the benefits of utilizing the centroid—or center of mass—over the geometric center, as the centroid accounts for the increased density on the right side of the space. In this example, it is possible to see that leg thickness appears to be driving similarity judgments, with thick-legged spiders in the lower left of the space and thin-legged spiders in the upper right of the space. The centroid more aptly depicts thick-legged spiders as being less prototypical for this particular space of spider images. The most “unique” item in the space (blue square)—the spider with the largest average pairwise distance from all other spiders—can be seen in contrast to the least unique spider (blue circle), which has the smallest average pairwise distance. Finally, the most central (red diamond) and most peripheral (red hexagon) spiders are classified according to their distance from the centroid (red circle)

Within the results (provided on this project’s Open Science Framework page), a metric of centrality is provided for each threat image in each of the MDS solutions (Fig. 4). Centrality represents a hypothetical measure of “prototypicality” in the context of the set of images being rated, where the more central an object is within the set, the more similar it is to a hypothetical prototypical object. To calculate centrality, we first calculated the center of mass (centroid) of the space of objects for each MDS solution by averaging each of the coordinate dimensions across all objects (see Table 2 for animate and Table 3 for inanimate objects). We believe this approach to designating the center of the MDS space—as opposed to calculating the geometric center, for instance—is superior in the context of psychological data because it places outlier objects in the periphery of the space. Consider the MDS space of spiders (Fig. 4), where there is a continuum of thick-legged to thin-legged spiders. Spiders with thinner legs outnumber spiders with thicker legs, and while the geometric center of this space would reside equidistant between all objects, this would not give any consideration to the relatively higher density of stimuli on the right side (thinner legs) of the space. The centroid of the space, however, does take into account the relative densities within the space. In this example, the centroid would exist farther toward the right side of the space than the geometric center, resulting in the spider images with thinner legs being more central than the spiders with thicker legs.

**Table 2:** Aggregate MDS space Euclidean distances between group centroids for animate threatening/nonthreatening object pairs

Animate		
Threatening	Nonthreatening	Distance
Wasp, hornet	Bee, fly	0.236837
Shark	Whale, dolphin	0.262031
Wolf, hyena	Dog	0.270264
Tiger, lion	Cat	0.336725
Alligator, crocodile	Lizard, turtle	0.365866
Spider	Ant	0.374783
Snake	Caterpillar, worm	0.509751
Scorpion	Stink bug, beetle	0.599617

**Table 3:** Aggregate MDS space Euclidean distances between group centroids for inanimate threatening/nonthreatening object pairs

Inanimate		
Threatening	Nonthreatening	Distance
Landmine	Weights	0.300276
Syringe	Knitting/sewing needle	0.348995
Taser	Electric razor, mobile recorder	0.392049
Baseball bat, Baton	Rolling pin, bowling pin, Umbrella	0.408211
Long gun	Paintball gun, water gun	0.425692
Knife, cleaver	Kitchen utensils	0.475145
Pistol	Hairdryer, toy gun	0.568287
Grenade	Ornaments, lime, juice container, perfume bottle	0.641542

### Item uniqueness scoring

In addition to centrality scores, the outputs include a metric of “uniqueness” for each object in each MDS solution (Fig. 4). Uniqueness is a metric that is independent of centrality. Rather than relating each exemplar to the centroid of the space, uniqueness instead relates each threat image to every other threat image in the space. To achieve this—for each image in the space—the mean pairwise distance between a threat image and every other threat image in the space is calculated. The most unique threat image in the space will be the one with the greatest average pairwise distance between it and any other image in the space. The least unique threat image will be the image with the smallest average pairwise distance in the total set of pairwise distances in the space.

### The ThreatSim database

To illustrate how the categories within the classes relate to each other in terms of visual similarity, we created plots showing the first two dimensions of the MDS space of all animate (Fig. 5) and inanimate (Fig. 6) objects in each category. On the plots, each category is represented by the image of the most central object in that category. Both plots show visually similar nonthreatening counterparts to the threatening images, indicating that the ThreatSim database can be used in future research as a reliable source of threatening and nonthreatening objects rated for similarity. In terms of the animate class, there appear to be four clusters: animals living in or near water, snake-like animals, land mammals, and arthropods. For the inanimate class, round objects seem to occupy the lower right quadrant and elongated objects the lower left. Also, with the exception of the hairdryer and the Nerf gun (both of which look like weapons), clearly threatening objects seem to cluster north of 0 on the *y*-axis, with weapons on the left and explosives on the right.

### MDS space for animate objects

**Fig. 5:**
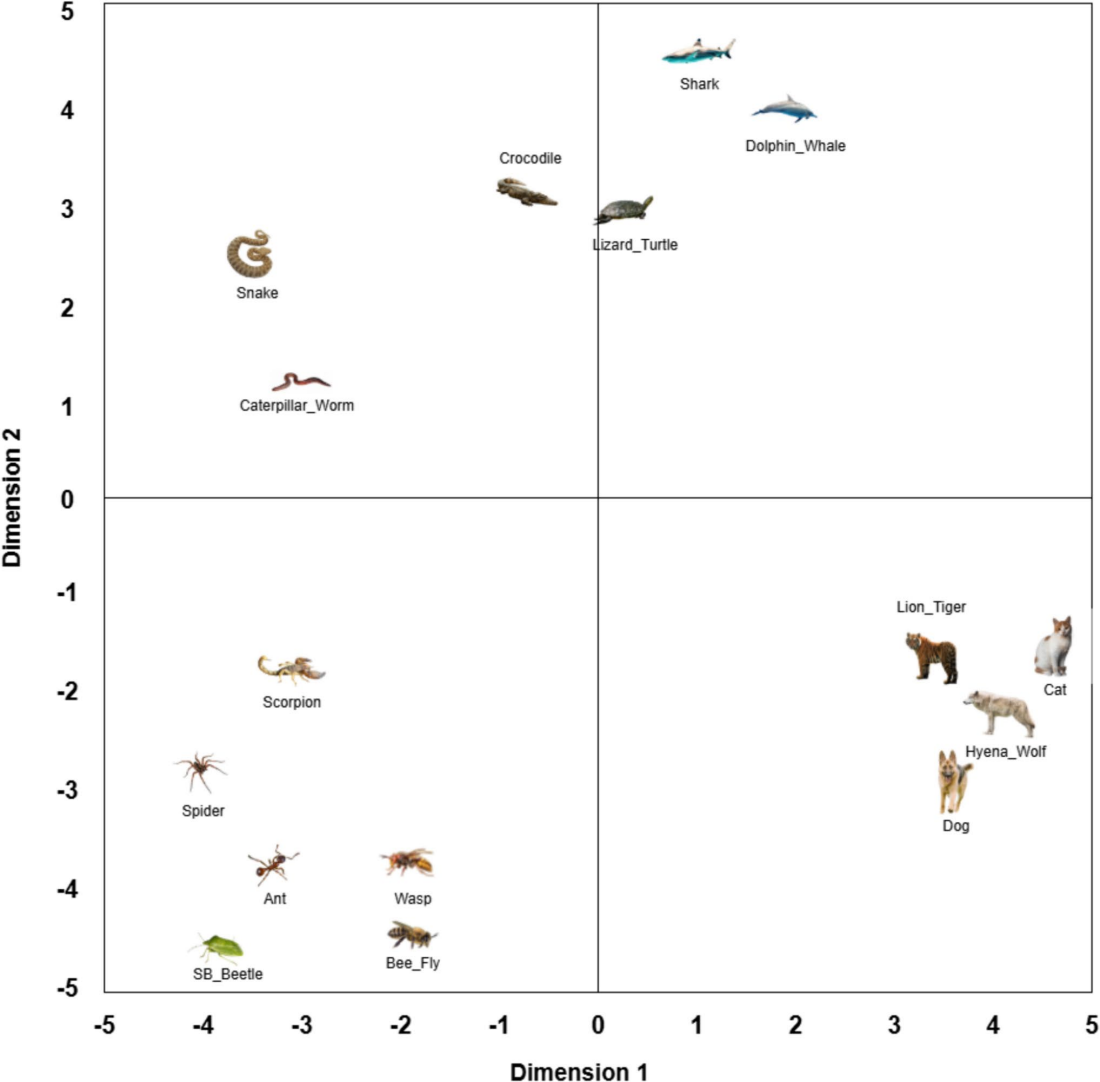
The first two dimensions of the MDS space for all animate objects in each category. Each image is the most central object in its given category, plotted in the location of that category’s centroid

#### MDS space for inanimate objects

**Fig. 6:**
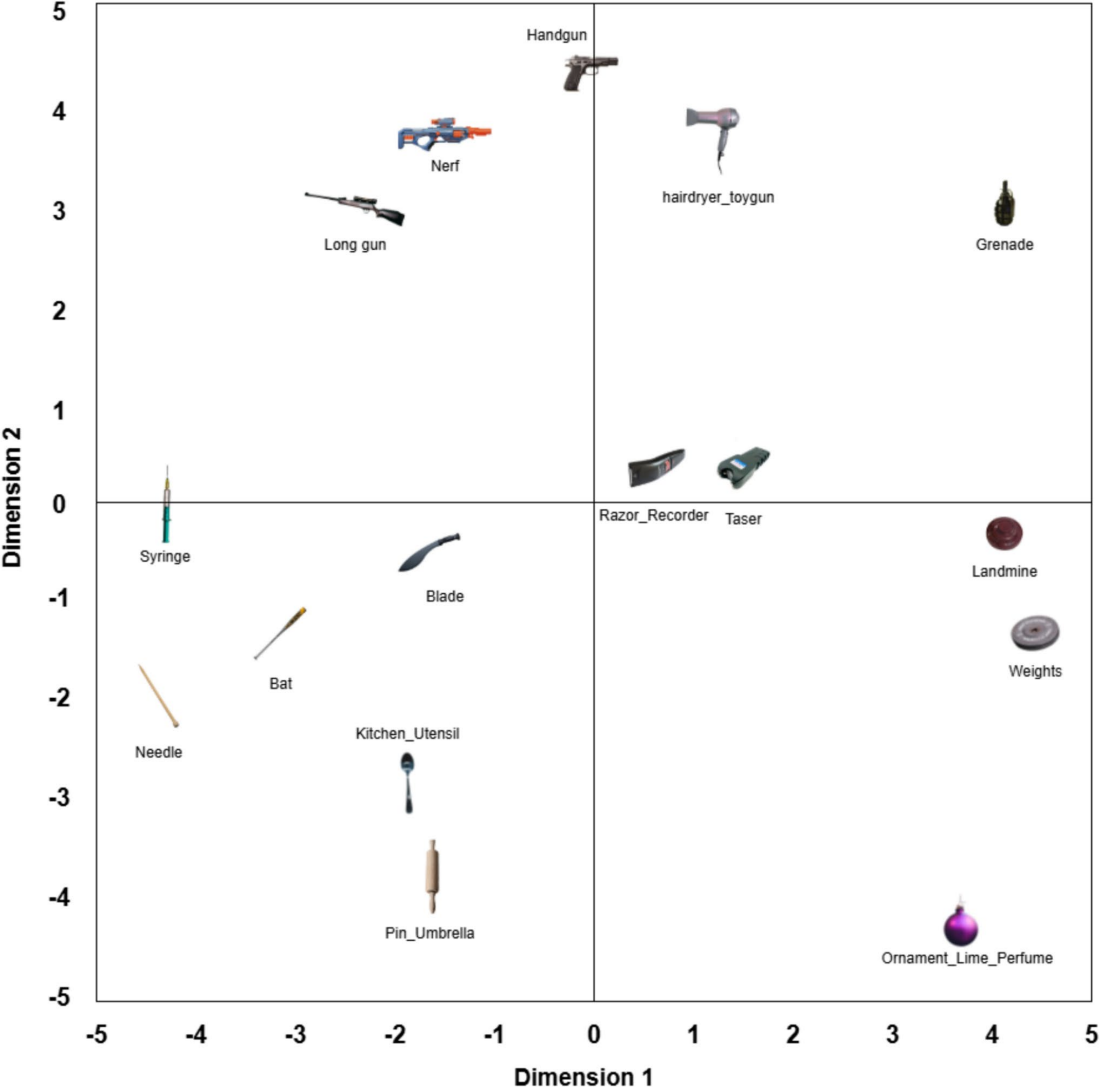
The first two dimensions of the MDS space for all inanimate objects in each category. Each image is the most central object in its given category, plotted in the location of that category’s centroid

### Summary of results

Put simply, our analysis shows how people perceive visual similarity between threatening and nonthreatening objects. Using MDS, we mapped the images onto a virtual plane, where the distance between items reflects how similar people thought they looked—closer items were judged to resemble each other more. The axes in these plots do not represent fixed physical features, such as size or color, but rather combinations of features that participants used, either consciously or unconsciously, to judge similarity. For instance, in the animate image space, we observed clusters of aquatic animals (e.g., sharks and dolphins) and arthropods (e.g., spiders and ants), suggesting that participants categorized images on the basis of biological or shape-related features. This spatial arrangement enables researchers to select threat and control images that are visually matched, thereby improving the control of future experiments.

## Discussion

Research on the effects of threats on visual attention is a fertile area in which a methodological shift is taking place. Recently, researchers have focused their efforts on improving stimulus selection and on using open and reproducible methods. New databases and paradigms (e.g., Bonin et al., 2014; Hedger et al., 2019; Zsido et al., 2019, 2024) have been developed to address a variety of concerns with past studies, with the aim of improving ecological validity, standardization, and transparency. The number of image databases available to researchers is growing, but many only partially meet the needs of the research field. A good image database for threat research must be both specific (including threatening content) and broad (including a large number of categories, animate and inanimate objects alike). The most important of these, which previous studies have tended to either control for or manipulate, is visual similarity. To our knowledge, no threat database has directly evaluated stimulus similarity. Furthermore, and related to this evaluation process, no previous database has relied on input from naïve participants to guide the selection of categories evaluated as threatening. Therefore, in the present study, we sought to create a novel threat image database that includes a large number of categories and objects that people consider threatening (coupled with those that they consider visually similar but not threatening), and to have those items rated for visual similarity. For this purpose, we used SpAM to collect similarity ratings for all the objects in our database, and multidimensional scaling was used to provide similarity models of those data. The result is an image database with 32 categories and 640 objects in total, containing an equal number of animate and inanimate categories and of threatening and nonthreatening ones, all rated for visual similarity. Another strength of the database is that the similarity ratings were collected from a sample of participants from different cultural backgrounds (i.e., Hungary and the USA). This diversity is important because perceptions of threat and visual similarity can be influenced by a person’s cultural background and experiences, such as their familiarity with specific objects or symbolic associations. Drawing on participants from multiple cultural contexts provides greater certainty regarding the quality of the observations (by virtue of averaging over a larger number of people), and increases the diversity of perceptions represented in our dataset.

Our database is a ready-to-use collection of freely available (copyright-free) images. Scree plots indicate that for most categories, the appropriate number of dimensions is greater than 2, based on a heuristic look at the “elbow” in the plot, which indicates substantial improvement in fit beyond that point. However, given the exploratory nature of this study, we generated two-dimensional plots because they allow for easy visual inspection of the space. These plots can greatly assist future users of the database in deciding which objects to include in their study. In terms of categories, these plots can serve as evidence of the visual similarity between a threatening and a nonthreatening category, allowing researchers to investigate the effects of emotional and visual features of threats on visual attentional or working memory processes. In addition, information about the diversity of each category (i.e., the category plots) informs researchers about the differences in exemplars within that category. This can be useful for selecting either very similar or very dissimilar exemplars of the category, opening up the possibility of studying the effect of semantic category and visual appearance.

It should be noted that, unlike previous databases, we focused on similarity ratings rather than emotional dimensions. This is not necessarily a shortcoming, as we only included categories based on the input of naïve participants, so relying on specific emotional dimensions was not as crucial in our view. However, collecting item-specific ratings on emotional dimensions (such as arousal or valence) may increase the usefulness of the database by allowing future research to examine finer-grained differences and associations between visual processing biases and these emotional dimensions.

We designed the ThreatSim database to be flexible and applicable to a wide range of experimental paradigms. Researchers studying visual attention, perception, emotion, or threat detection can use this resource to carefully match threatening and nonthreatening stimuli in terms of visual similarity, which is a feature that has been lacking in previous stimulus sets. Studies using dot-probe tasks, eye tracking, or visual search can now control for mid-level visual features when comparing threatening and emotionally neutral stimuli. In cognitive neuroscience, these images could be used to isolate neural responses to threat-relevant content, eliminating (or at least reducing) confounding factors related to perceptual differences. The database is also valuable for developmental and cross-cultural studies in which researchers may wish to investigate whether threat detection biases are affected by learning, familiarity, or cultural background. Moreover, the centrality and uniqueness metrics provided with the database allow for greater experimental control. Depending on the hypothesis being tested, researchers can select more “prototypical” or more “distinctive” items within a category. We believe this makes ThreatSim particularly well suited to research investigating semantic categories, categorical boundaries, or attentional tuning.

The addition of centroid distance and distributional metrics (prototypicality and uniqueness) enhances the practical utility of the database even further. These values provide researchers with a systematic approach to selecting stimuli with specific similarity profiles, such as closely matched threat–neutral pairs or items that vary in category centrality. This enables more controlled investigations into visual attention, perception, or memory. These similarity ratings can also be used to improve the design of experiments in visual search paradigms. For instance, Hout and Goldinger (2015) demonstrated that when target templates were imprecise or contaminated by irrelevant features, both attentional guidance and decision-making during the search process were impaired. Having access to quantified similarity relationships, such as those provided by ThreatSim, could enable researchers to manipulate template precision systematically and explore the effect of visual similarity between targets and distractors on search performance.

One limitation of the database is that the semantic relationship between threatening and nonthreatening pairs is unclear. While most pairs in the animate set belong to the same taxonomic category (e.g., wasps and bees), items in the inanimate set are more functionally or visually matched than semantically equivalent (e.g., grenades and ornaments). This asymmetry reflects the nature of human-made categories, which are less taxonomically constrained than biological categories. It also limits the interpretability of direct comparisons between animate and inanimate stimuli. However, as the animate and inanimate image sets were developed and analyzed independently, researchers can choose to work within a single class depending on their specific goals.

A second limitation of the dataset is that some threatening items are not always paired with their nonthreatening counterparts. For instance, within the inanimate stimulus space, a grenade may appear closer to visually similar objects, such as weights or razors, than to its designated pair partner (e.g., a perfume bottle). This reflects the complexity of human similarity judgments and highlights the importance of relying on empirical data rather than assumed pairings. Rather than undermining the database, this nuance enhances its utility; researchers can select stimulus pairs according to their proximity in MDS space, enabling more precise control over (or manipulation of) visual similarity in experiments. Studies on threat generalization or training in high-stakes visual categorization tasks (e.g., airport screening) could benefit from selecting pairs that span different levels of similarity, for instance.

A third limitation is that the “threatening” and “nonthreatening” labels in ThreatSim were derived from an independent survey in which participants nominated objects for each category. While this approach ensures that the stimuli reflect lay perceptions, it also means that some “nonthreatening” items, such as toy or water guns, may still evoke heightened arousal due to their visual resemblance to genuinely threatening objects. This nuance could be advantageous for researchers studying how visual and emotional properties interact to shape threat responses. However, it also suggests an area for future research: obtaining comprehensive ratings of perceived threat level and arousal for all stimuli, in order to complement the visual similarity data presented here.

In summary, we believe that this threat database is a resource that will enable scientists interested in threat research and other fields involving visual attention and working memory to obtain the best possible experimental control in situations where real-world stimuli of animate and inanimate objects (or threats and neutral controls) are required. Compared with previous databases, this tool provides images that can be evaluated by anyone and should allow researchers to increase the confidence in their results. This is also a potential step toward increasing the reproducibility of results, drawing conclusions with greater generalizability, and thus advancing our understanding of the processes underlying threat perception, detection, attention, and memory biases.

## Data Availability

The datasets generated and analyzed during the current study are available in the Open Science Framework repository, https://osf.io/cmtdw/.

## Code availability

The code used for data analysis is available in the Open Science Framework repository, https://osf.io/cmtdw/.
